# Stroke survivors show an overestimation of their on-road driving performance

**DOI:** 10.1186/s42466-025-00406-y

**Published:** 2025-07-31

**Authors:** Daniel A. Schlueter, Gernot Horstmann, Kim L. Austerschmidt, Jessica Koenig, Maximilian Flieger, Thomas Beblo, Martin Driessen, Wolf Schäbitz, Max Toepper

**Affiliations:** 1 Department of Psychiatry and Psychotherapy, Evangelisches Klinikum Bethel (EvKB), University Medical Center OWL, Bielefeld University, Remterweg 69-71, 33617 Bielefeld, Germany; 2 Department of Neurology, Evangelisches Klinikum Bethel (EvKB), University Medical Center OWL, Bielefeld University, Burgsteig 13, 33617 Bielefeld, Germany; 3 Department of Neuro-Cognitive Psychology, Bielefeld University, Universitätsstraße 25, 33615 Bielefeld, Germany

**Keywords:** Stroke, Fitness to drive, Driving skills, Self-assessment, Self-rating

## Abstract

**Objectives:**

Strokes are often accompanied by physical and cognitive impairments affecting driving safety. After the recommended period of abstinence from driving, the patient must decide whether his or her driving safety is still impaired, which requires a valid self-assessment of the own driving skills. At present, it is uncertain whether stroke survivors are able to provide a valid self-assessment**.**

**Methods:**

12 stroke patients and 17 healthy controls participated in this prospective longitudinal on-road study. All participants underwent repeated neuropsychological and standardized on-road assessment at 4-month intervals (2 and 6 months after the stroke in the patient group). Statistical analyses included repeated measures ANOVA, group comparisons and correlation analyses.

**Results:**

Our results revealed that in stroke survivors compared to healthy drivers, the validity of self-assessment (VSA) of the own on-road driving performance is impaired in the direction of overestimation (at both time points). In addition, the VSA of stroke survivors at second time point correlated with driving-relevant cognitive and non-cognitive measures.

**Discussion:**

Our results suggest that the VSA of the own driving competence is impaired after stroke. Other than expected, the differences between stroke survivors and healthy drivers did not disappear within the 4-months-interval. Consequently, an impaired VSA in stroke survivors must be considered before deciding to let them drive again.

## Introduction

German road traffic regulations require every driver to ensure safe driving by assessing his or her own fitness to drive and taking preventive action if there are any conditions that may affect driving safety. In particular, this applies to drivers suffering from neurological diseases such as stroke. Stroke is a heterogeneous neurological disorder with focal neurological deficits due to cerebral damage, most commonly caused by the occlusion of a blood vessel (ischemic stroke) or, less commonly, by cerebral hemorrhage (hemorrhagic stroke). Its lifetime prevalence of around 3% in Germany [6] and a yearly incidence of over 1 million cases in the European union [32] make stroke one of the most frequent neurological diseases of our time. As the risk of stroke increases with age, we are seeing an increasing number of cases as the population of western industrialized countries ages [30]. The range of stroke-related impairments extends from barely noticed and rapidly recovering cognitive (e.g. language, memory or attention deficits), physical (e.g. paresis) or psychiatric (e.g. post-stroke depression) symptoms to severe permanent impairments. Due to this heterogeneity, which is influenced by the type, location and severity of the stroke, the impairment itself and the potential for recovery are highly individual and cannot be accurately predicted. Certainly, however, these impairments often have a serious impact on driving safety. Meta-analytic data show that 46% of stroke patients fail a practical driving test and even mild strokes are associated with twice as many driving errors during simulated driving [8]. In addition, there is evidence for an increased risk of accidents. Noteworthy, impairments and accident risk are highly individual, depending on the type, location and severity of the stroke, as well as the time since the event [28].

Despite this, or perhaps because of it, neither legislation nor guidelines for assessing fitness to drive give specific recommendations on when it is possible to drive again after a stroke. In the majority of cases, the doctor in charge will inform the patient to take a break from driving for three or six months depending on functional deficits and the risk of stroke recurrence. In the months following the stroke, some of the impairments may recover spontaneously and so may fitness to drive [28]. It is assumed that around one third of stroke survivors can resume driving with little or no training. However, a third of patients need intensive training before they can continue driving, while the last third of drivers may never be able to drive again [1]. This is problematic insofar as in 50% of stroke survivors, regaining the ability to drive is the main therapeutic goal after a stroke because of its high relevance for mobility and participation in everyday life [8, 17]. On the other hand, only 10% of drivers who resume driving after a stroke have undergone a formal driving assessment beforehand [8]. Consequently, this results in a large number of stroke survivors who continue driving despite driving-relevant dysfunctions. For these drivers, a valid self-assessment and efficient regulatory processes appear to be highly relevant to compensate for deficits and ensure driving safety.

Unfortunately, little is known about the validity of self-assessment in stroke survivors. The few previous studies on this topic found that stroke survivors tend to overestimate their ability to drive [19, 29], which appears to be associated with an impaired awareness of cognitive deficits [13, 16]. In addition, poor awareness of stroke-related impairments was reported to be related with poorer rehabilitation success [13].

### Rational of the study

Taken together, there is little evidence for the relationship between on-road driving skills and the validity of self-assessment (VSA) in stroke survivors and this particularly applies to the recovery of VSA in the months following a stroke. In the current prospective longitudinal study, we therefore examined the VSA of stroke survivors and healthy drivers by comparing the subjective ratings of these groups with the objective ratings provided by a driving expert at two time points (two and six months after the stroke in stroke survivors and at a 4-months interval in healthy controls, respectively).

Taking into account the above considerations, we expect that the stroke group will have a more invalid self-assessment of their on-road driving performance compared to the control group in the direction of overestimation. This difference should decrease over time. Secondly, we expect that the VSA of stroke survivors and the recovery of their VSA over time is associated with cognitive recovery.

## Methods

### Participants

Initially, we recruited 20 older German drivers from the general population via local newspaper articles and 30 stroke patients from the stroke unit of Evangelisches Klinikum Bethel (University Hospital OWL, Bielefeld University, Department of Neurology). Inclusion criteria for healthy participants were a valid drivers’ license, being a currently active driver and having an age of at least 50 years. Exclusion criteria for healthy participants involved current diseases relevant for fitness to drive (i.e., recent craniocerebral traumas, strokes, heart attacks, epileptic seizures, dementia, acute dizziness, diabetes with severe metabolic disturbances, and other conditions that are at risk of sudden unconsciousness; (i.e., recent craniocerebral traumas, strokes, heart attacks, epileptic seizures, dementia, acute dizziness, diabetes with severe metabolic disturbances, and other conditions that are at risk of sudden unconsciousness; [12]), current mental disorders as identified by the screening questions from the structured clinical interview for DSM-IV (i.e., substance abuse, psychosis, moderate to severe depression; [34]) as well as the intake of medication with a severe influence on driving fitness (category III) determined using the Driving under the Influence of Drugs, Alcohol and Medicines (DRUID) checklist [11]. Due to a withdrawal of interest, one participant had to be excluded from the study. In addition, two participants were excluded from analysis due to missing or invalid data. The final dataset included 17 healthy older drivers (8 females, 9 males) with a mean age of 75.1 years (*SD* = 6.4; range = 65–87) and a mean school education of 11.1 years (*SD* = 2.1).

Inclusion criteria for the stroke patients were an acute ischemic stroke confirmed by imaging two month before the first assessment with regression of severe clinical symptoms within eight weeks, which according to the assessment guidelines for fitness to drive would have automatically led to a loss of fitness to drive. Additional inclusion criteria were a valid drivers’ license, being an active driver before the stroke and having an age of at least 50 years. Exclusion criteria for stroke patients involved the same current diseases and conditions relevant for fitness to drive as for healthy participants, except for the stroke.

Due to withdrawal of interest, acute somatic complaints in the further course of the study, fear of the on-road assessment, mental health issues, 13 patients had to be excluded from the study. Additionally, five patients were excluded due to missing or invalid data. The final dataset included 12 stroke patients (2 females, 10 males) with a mean age of 69.6 years (*SD* = 9.0, range = 52–83) and a mean school education of 10.6 years (*SD* = 1.9). While a proportion (25%) of the patients exhibited symptoms of severe stroke at admission, data for the overall sample of stroke survivors point towards a mild impairment. An overview of the lesion locations is displayed in Table 1. Clinical stroke-related data of the patient sample are displayed in Table 2. Sample characteristics are displayed in Table 3.

**Table 1:** Lesion locations of stroke patients included in the present study (*n* = 12)

	*n* (%)
Arteria cerebri anterior, left	1 (8.3%)
Arteria cerebri media, left	5 (41.7%)
Arteria cerebri posterior, left	1 (8.3%)
Arteria cerebri posterior, right	2 (16.7%)
Corona radiata, right	1 (8.3%)
Fragmented pons infarct	1 (8.3%)
Border zone infarct	1 (8.3%)

**Table 2:** Stroke-related clinical data of the patients included in the present study (*n* = 12)

NIHSS score at admission *M* (*SD*)	–	4.8 (6.0)
NIHSS score at admission range	–	0–19
NIHSS score in time course *M* (*SD*)	–	2.3 (1.9)
NIHSS score in time course range	–	0–6
mRS score at admission *M* (*SD*)	–	1.6 (1.4)
mRS score at admission range	–	0–4
mRS score at discharge *M* (*SD*)	–	0.6 (0.5)
mRS score at discharge range	–	0–1
Rehabilitative treatment (*n*)^a^	–	11
Duration of rehabilitative treatment *M* (*SD*)^b^	–	15.4 (5.4)
Duration of rehabilitative treatment range^b^	–	0–20

*M* = mean; *SD* = standard deviation; *NIHSS* = National Institutes of Health Stroke Scale; *mRS* = modified Rankin scale; ^a^number of patients who received rehabilitative treatment; ^b^in days

**Table 3:** Sample characteristics for healthy older adults (*n* = 17) and stroke patients (*n* = 12) including demographic and driving-relevant data

	Healthy	Stroke
*N*	17	12
Sex (female/male)	8/9	2/10^+^
Age *M* (*SD*)^a^	75.1 (6.4)	69.6 (9.0)
Age range^a^	65–87	52–83
School education *M* (*SD*)^a^	11.1 (2.1)	10.6 (1.9)
School education range^a^	8–13	8 – 13
Number of prescribed drugs *M* (*SD*)	2.9 (2.1)	6.8 (3.0)^**^
Number of prescribed drugs range	1–6	3–10
MMSE_T1_ *M* (*SD*)	28.8 (1.4)	27.8 (2.1)
MMSE_T1_ range	27–30	22–30
Annual mileage in 1000 km *M* (*SD*)^b^	13.4 (9.3)	9.2 (5.1)
Annual mileage in 1000 km range^*b*^	2.5–33.6	3.0–20.0
Years of having a driver’s license *M* (*SD*)	55.1 (5.5)	50.3 (8.5)
Years of having a driver’s license	47–63	33–64
Unfit to drive at T1/T2	0 (0%)/0 (0%)	4 (33,3%)^*^/2 (16.7%)^+^
Self-rated on-road driving performance_T1_^c^, *M* (*SD*), [range]	2.6 (0.8) [1.5–4.5]	2.3 (0.7) [1.5–4.0]
Self-rated on-road driving performance_T2_^c^, *M* (*SD*), [range]	2.6 (0.7) [2.0–4.5]	2.3 (0.5) [2.0–3.0]
Expert-rated on-road driving performance_T1_^c^, *M* (*SD*), [range]	2.1 (1.0) [1.0–4.0]	3.1 (1.4) [1.5–5.0]^*^
Expert-rated on-road driving performance_T2_^c^, *M* (*SD*), [range]	2.1 (0.8) [1.5–3.5]	2.7 (1.3) [1.5–5.0]
VSA_T1_ *M* (*SD*), [range]	− 0.6 (1.1) [− 2.5–2.0]	0.7 (1.1) [− 0.5–3.0]^**^
VSA_T2_ *M* (*SD*), [range]	− 0.5 (0.8) [− 2.0–1.0]	0.5 (1.2) [− 1.0–3.0]^*^
Overestimators T1/T2	2 (11.8%)/2 (11.8%)	5 (41.7%)/4 (33.3%)
Valid estimators T1/T2	7 (41.2%)/7 (41.2%)	7 (58.3%)/7 (58.3%)
Underestimators T1/T2	8 (47.0%)/8 (47.0%)	0 (0%) 1 (8.3%)

*M* = mean; *SD* = standard deviation; *MMSE* = Mini-Mental State Examination; *TRIP* = Test Ride for Investigating Practical fitness-to-drive protocol; *T1* = first time point (2 month after stroke in the patient group); *T2* = second time point (6 month after stroke in the patient group) VSA = Validity of self-assessment; ^a^in years; ^b^in kilometers; ^c^11-point-Likert-scale; ^+^*p* < 0.10; *p < 0.05; **p < 0.01

### Study protocol

All participants underwent the same study protocol involving four experimental sessions, one feedback session and a telephone interview. Before study start, all participants were interviewed on the telephone to collect basic data and to screen for inclusion and exclusion criteria in the healthy group. The first assessment time point (two month after stroke in the patient group) consisted of two sessions: The first session involved a neuropsychological assessment of various cognitive functions and the collection of driving-relevant data. This procedure was repeated with all participants after four months (third session).

The second session involved a standardized on-road assessment that was repeated with all participants after four months (fourth session). In a fifth session, participants were given detailed feedback regarding their cognitive performance as well as their on-road driving performance. The study protocol was in line with the Declaration of Helsinki and approved by the Ethical Review Board of University of Münster. All participants joined voluntarily in the study and gave informed written consent.

### Measures

#### Neuropsychological assessment

Neuropsychological examination involved the assessment of different cognitive sub functions such as memory, attention, executive functions and spatial abilities. The Mini Mental Status Examination [10] was used as a screening of global cognition. Verbal episodic memory was assessed with the German version of the Rey auditory verbal learning test [14]. Subtests of the Consortium to Establish a Registry for Alzheimer’s Disease [20] were used to examine figural episodic memory (Constructional Praxis II) and semantic memory (15-items version of the Boston Naming Test; BNT). Non-verbal episodic memory was also assessed by the Rey-Osterrieth-Complex-Figure-Test [21, 25]. Furthermore, a semantic fluency task (animal fluency; [2]) was applied. Attentional functioning was assessed with the Trail Making Test part A (psychomotor speed; [24]), a digit span task (attention capacity, Wechsler Adult Intelligence Scale; WAIS-IV; [33]) and four subtests (alertness, divided attention, visual field and visual scanning) of the Testbatterie zur Aufmerksamkeitsprüfung (TAP; [35]). Executive functions such as verbal fluency as well as verbal and nonverbal cognitive flexibility were measured with a phonemic fluency task (RWT: S-Woerter; [2]), a reaction change task (subtest flexibility; TAP; [35]), a phonemic category change task (RWT: H-T-Woerter; [2]) and part B of the Trail Making Test (TMT-B; [24]). Additionally, a digit word transformation task [15] and a go/no-go task (subtest Go/Nogo; TAP; [35]) were used as well as a German version of the Stroop Color Word Test [4] assessing inhibitory functioning. Verbal working memory was examined by a digit span task backward (WAIS-IV). Spatial abilities were assessed by using a CERAD sub test (Constructional Praxis I), the clock drawing test and the Snellgrove Maze Task [31]. Additionally, personality was assessed by the German version of the Big Five Inventory (NEO-FFI; [5]).

#### On-road driving assessment

On-road driving assessments were conducted on a constant weekday between 8:30 a.m. and 3:30 p.m. on an 18-km standardized route. Driving school vehicles were equipped with an automatic or manual transmission according to participants’ private car use. To ensure a sufficient difficulty of the route, experts planned the course taking into account accident blackspots (i.e., traffic locations where in the last years accidents had been frequently registered in the city’s official accident register). A driving instructor and a driving expert – both completely blinded to all of the participants’ characteristics (e.g., age, test scores, etc.) – accompanied the on-road assessments. On-road driving performance was evaluated by the driving expert using the German version of the well-established “Test Ride for Investigation Practical fitness-to-drive” protocol (TRIP; [7, 22]). As provided in the TRIP, the expert rated fitness to drive on a 4-point scale with the categories 1 = “fit to drive”, 2 = “fit to drive, but driving training proposed”, 3 = “not fit to drive, but possibly fit to drive after driving training”, or 4 = “unfit to drive despite driving training”. For further analyses, this rating was dichotomized into drivers who were currently fit to drive (1 and 2) and drivers who were currently unfit to drive (3 and 4). This classification served as binary criterion for fitness to drive. Moreover, the driving expert rated the on-road driving performance on an 11-point- Likert scale ranging in steps of 0.5 from 1 = “very good” to 6 = “unsatisfactory”.

Neither the starting time of the on-road assessment, nor the type of transmission system, vehicle type, weather conditions (dry road vs rain/wet) or familiarity with the route were significantly associated with driving skills (all *p* > 0.05).

#### Self-assessment of driving skills

Self-assessment of the own driving ability was measured at three timepoints with the same scales. The first rating referred to the general driving ability and was made before study participation during the telephone interview using an 11-point- Likert scale ranging in steps of 0.5 from 1 = “very good” to 6 = “unsatisfactory”. The second and third rating referred to the on-road driving performances and was made directly after the on-road tests at T1 and T2, also using an 11-point- Likert scale ranging in steps of 0.5 from 1 = “very good” to 6 = “unsatisfactory”.

#### Feedback session

At the end of the study, all participants were given detailed feedback on their cognitive performances in both neuropsychological assessment sessions as well as on the results of their on-road driving performance and fitness to drive. In addition, all participants received information material (e.g. driver safety training). The data collected in the feedback session were not part of the analyses for the current manuscript.

### Data analyses

Data analyses included descriptive statistics of the total sample, group comparisons, repeated measures ANOVA as well as correlation analyses. Due to small and unequal group sizes (requirements for parametric tests were violated for the majority of the variables), demographics of groups were compared with non-parametric Mann–Whitney-U-Test und Chi^2^ test.

To examine the VSA, we first calculated the difference between self- and expert ratings (expert rating minus self-rating). Positive values represented overestimation with higher values reflecting a higher degree of overestimation. Vice versa, negative values reflected underestimation with lower values reflecting a higher degree of underestimation. Values between 0.5 and -0.5 were defined as an adequate self-rating. Values could range between 5 (indicating the highest degree of overestimation) and -5 (indicating the highest degree of underestimation) with 0 indicating the most valid estimation. Since all requirements for parametric testing in the VSA variable were met, a 2 × 2 repeated measures ANOVA was calculated to test for differences in VSA between groups and within groups between the two time points (T1 and T2). Exploratory, we then computed non-parametric correlations (Kendall’s Tau) between the VSA and demographic variables (e.g. age, gender, education), clinical data (e.g. National Institutes of Health Stroke Scale (NIHSS) score and modified Rankin scale (mRS) score) and cognitive performance (neuropsychological test scores and driving-relevant variables (e.g. mileage). Data were analyzed with IBM SPSS Statistics 25.0.2 (SPSS Inc.). All levels of significance were α ≤ 0.05 and tests were two-tailed.

## Results

### Demographic and driving-related data

Sub group comparisons between healthy older drivers (*n* = 17) and stroke survivors (*n* = 12) did not reveal significant differences in age (*U* = 67.0, *p* = 0.128), years of school education (*U* = 74.5, *p* = 0.556), years of having a driver’s license (*U* = 69.5, *p* = 0.152), MMSE score (*U* = 65.5, *p* = 0.107), annual mileage (*U* = 81.0, *p* = 0.370), self-rated driving performance at T1 (*U* = 79.5, *p* = 0.325) and T2 (*U* = 66.0, *p* = 0.117) as well as the expert-rated driving performance at T2 (*U* = 75.0, *p* = 0.245). However, the number of prescribed drugs was significantly higher in stroke group (*U* = 32.5, *p* = 0.001), while the expert-rated driving performance at T1 was significantly lower (*U* = 57.5, *p* = 0.048). Additionally the number of unfit drivers in the stroke group was significantly higher at T1 (*Chi*^*2*^(1) = 6.573, *p* = 0.010) and marginally higher at T2 (*Chi*^*2*^(1) = 2.872, *p* = 0.090) and so was the number of male participants in the stroke group (*Chi*^*2*^(1)= 2.876, *p* = 0.090).

Calculation of differences between expert and self-rating at T1 revealed five (41.7%) overestimators in the stroke group as well as seven valid estimators (58.3%) and no underestimators. In the group of healthy drivers, analyses identified two overestimators (11.8%), seven valid estimators (42.2%) and eight underestimators (47.0%). At T2, analyses revealed four overestimators (33.3%) in the stroke group, seven valid estimators (58.3%) and one underestimator (8.3%), In the healthy group, we found two overestimators (11.8%), seven valid estimators (41.2%) and eight underestimators (47.0%).

### Association between self- and expert assessment

Correlation analyses revealed a significant strong positive correlation between general self-rating of the own driving ability before study participation and the self-rating of the own driving ability directly after the on-road driving assessment at T1 in the stroke group (*r* = 0.702, *p* < 0.011), but not in the healthy group (*r* = 0.328, *p* = 0.198). Between self- and expert rating directly after the on-road assessment at T1, a significant high positive correlation was observed in the stroke group (*r* = 0.622, *p* = 0.031), but not in the healthy group (*r* = 0.233, *p* = 0.369).

### Group level analysis of VSA over time

**Fig. 1:**
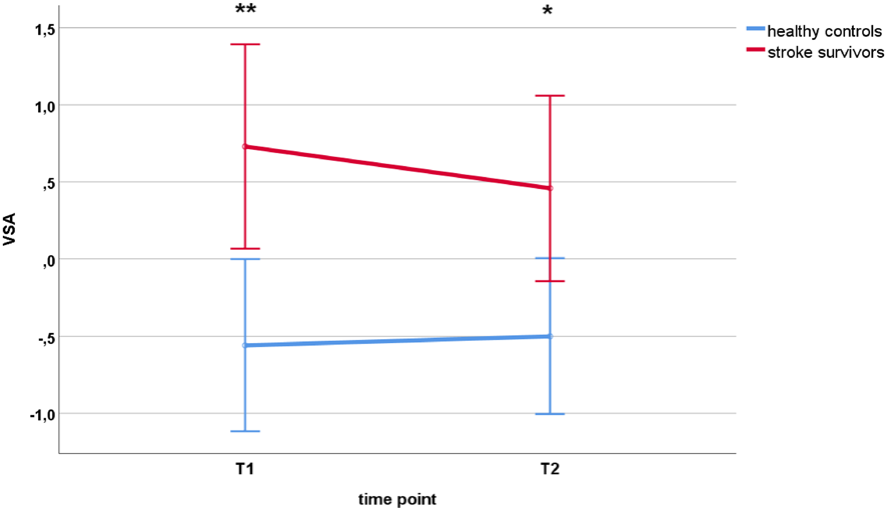
VSA of healthy controls and stroke survivors at first (T1) and second (T2) time point. Error bars represent 95% confidence interval. *T1* = first assessment time point (2 month after stroke in the stroke group); *T2* = second assessment time point (6 month after stroke in the stroke group); *VSA* = validity of self-assessment

The 2 (time point: T1, T2) × 2 (group: healthy, stroke) repeated measures ANOVA for VSA resulted in a significant main effect of group (*F*_(1,27)_ = 9.252, *p* = 0.005*)*. Neither the main effect of time point (*F*_(1,27)_ = 0.435, *p* = 0.515) was significant, nor was the time point x group interaction (*F*_(1,27)_ = 1.052, *p* = 0.314). Post-hoc t-tests revealed higher VSA values in the stroke group compared to the healthy group at T1 (*T*_(27)_ = − 3.050, *p* = 0.005) and at T2 (*T*_(27)_ = − 2.508, *p* = 0.018). Results are displayed in Fig. 1.

### Explorative correlations

Total scores of the National Institutes of Health Stroke Scale (NIHSS) and the modified Rankin Scale (mRS) at admission and discharge did not correlate significantly with VSA at T1 or T2, indicating that VSA was not associated with stroke severity and the degree of disabiliy in the current sample (probably due to the small sample size).

In the stroke group, non-parametric correlation analyses (Kendall’s Tau) showed significant strong correlations between VSA at T2 and cognitive flexibility as measured by the respective TAP subtest (*r* = 0.749, *p* = 0.009), interference speed (*r* = 0.643, *p* = 0.030) and accuracy (self-corrected errors; *r* = 0.742, *p* = 0.021) as measured by the Stroop Color Word Test, response inhibition as measured by median reaction times in the TAP subtest Go/Nogo (*r* = 0.618, *p* = 0.034), divided attention (auditory) as measured by the respective TAP subtest (*r* = 0.629, *p* = 0.014), median reaction times in the central (*r* = 0.686, *p* = 0.011) and omits in the left visual field (*r* = 0.667, *p* = 0.044) as measured by the respective TAP subtests, visuospatial abilities (CFT; *r* = − 744., *p* = 0.002) and non-verbal memory (CFM; *r* = − 0.593, *p* = 0.009) as measured by the respective ROCFT subtests and the personality factor openness (*r* = − 0.744, *p* = 0.001) as measured by the NEO-FFI.

## Discussion

In the current prospective longitudinal on-road study, we investigated the VSA of on-road driving performance and its association with cognitive and non-cognitive driving-relevant variables in stroke survivors and healthy older drivers over a period of four months. Results revealed significant group differences in the VSA between stroke survivors and healthy drivers. Thereby, stroke survivors showed an overestimation of the own on-road driving performance, whereas healthy drivers rated themselves more accurately. These group differences were observed at both time points. Other than expected, however, the VSA of the own on-road driving performance did not significantly change over time in none of the groups being indicated by a non-significant main effect of time and a non-significant group x time interaction. In addition, stroke survivors showed significant relations of overestimation with lower cognitive performance and with driving-relevant non-cognitive risk factors at the second time point.

The key finding of the current study is that the VSA was still impaired in patients at the second time point six months after the stroke. Although Fig. 1 suggests that group differences in VSA become slightly smaller over time, stroke survivors still had a significantly greater tendency than healthy drivers to overestimate their on-road driving performance. This appears to be insofar problematic that all drivers—independent from age or disease—must be able to validly judge their own driving competence as required by German traffic law. Anyway, stroke survivors may continue driving despite an invalid self-assessment, because the period of abstinence from driving, as recommended by the physician, had already passed at this point in time. Accordingly, stroke survivors may pose a risk even six months after the event.

The number of unfit drivers in the stroke group decreased from 33.3% two months after the stroke to 16.7% six months after the stroke. Nevertheless, no patient rated himself or herself as being unfit to drive at both time points indicating that subjective ratings are higher than objective ratings and that a partial recovery of driving fitness in the stroke group is not accompanied by a recovery of the VSA.

Another important finding is the association between VSA and a broad range of cognitive domains at the second time point such as attentional and executive functions, non-verbal memory, as well as between VSA and driving-relevant non-cognitive factors like personality. These findings are in line with prior research in healthy older drivers indicating that overestimation is associated with lower cognitive functioning [27] and that there seems to be a link between driving performance and somatic variables [9] as well as the personality factor openness [3].

From a diagnostic point of view, our results underline the need for an individual and valid post-stroke driving assessment, as a large proportion of stroke survivors is fit to drive shortly after the event and others need months before driving fitness has recovered. Due to an invalid self-assessment of the own driving skills, the decision when to be able to drive again must not be left to the patients. Importantly, the goal of post-stroke driving assessment is not only to restrict driving for many of the patients but also to allow driving in the absence of functional deficits.

From a therapeutic point of view, training programs could not only help to accelerate recovery from stroke-related cognitive, sensory or motor dysfunctions, but also improve the VSA.

## Conclusions and limitations

In the current study, we found that stroke survivors show an impaired self-assessment of their own driving performance as compared to healthy drivers both two and six months after the stroke. In particular, our results suggest that stroke survivors overestimate their own driving competence, even after the recommended period of abstinence from driving (three or six months). Importantly, we also observed a recovery of driving fitness in half of the unfit stroke patients within the time frame recommended by Marx et al. [18]. Although overestimation does not per se imply unfitness to drive, it can certainly be considered a risk factor for driving safety, as shown in previous research with healthy older drivers [27]. Moreover, an impaired self-assessment contradicts the conditions for fitness to drive as being anchored in German driving regulations.

Overall, these findings are of great relevance because they show that a stroke-related impairment of a valid driving self-assessment may exceed the recommended period of abstinence from driving and may thus entail serious consequences for safe road participation. Noteworthy, these results particularly apply for driving regulations in Germany. In other countries, regulations may differ with respect to the physicians’ obligation or right to impose or suggest a driving ban, the period of abstinence from driving or the type of assessment (e.g. medical examination, neuropsychological assessment).

Next to several strengths of the current work, some limitations have to be considered such as the small sample size, the unequal sex distribution (10 of 12 patients are male, which is why the results particularly apply to male stroke survivors) and the heterogeneity of strokes per se. In addition, we currently do not have causal evidence on which factors favor overestimation in stroke survivors and which factors lead to recovery of VSA. Noteworthy, mean and minimum age were lower in the stroke group than in the healthy group, which might have biased the results. However, given previous findings that revealed a positive association between age and overestimation, our results may rather underestimate the effects. [23, 26]. Future studies involving larger samples and longer observation periods (and/or more assessment points) should focus on how VSA changes post-stroke. 

## Data Availability

The data supporting the findings of this study are available on request from the corresponding author.
